# A High-Performance Portable Transient Electro-Magnetic Sensor for Unexploded Ordnance Detection

**DOI:** 10.3390/s17112651

**Published:** 2017-11-17

**Authors:** Haofeng Wang, Shudong Chen, Shuang Zhang, Zhiwen Yuan, Haiyang Zhang, Dong Fang, Jun Zhu

**Affiliations:** 1 College of Electronic Science and Engineering, Jilin University, Changchun 130012, China; whf@jlu.edu.cn (H.W.); chenshudong@jlu.edu.cn (S.C.); 2 Science and Technology on Near-Surface Detection Laboratory, Wuxi 214035, China; yuanzw2008@126.com (Z.Y.); zhyzhyzhy001@126.com (H.Z.); csdfangdong@163.com (D.F.)

**Keywords:** unexploded ordnance (UXO), portable system, transient electromagnetic sensor, sensor internal noise

## Abstract

Portable transient electromagnetic (TEM) systems can be well adapted to various terrains, including mountainous, woodland, and other complex terrains. They are widely used for the detection of unexploded ordnance (UXO). As the core component of the portable TEM system, the sensor is constructed with a transmitting coil and a receiving coil. Based on the primary field of the transmitting coil and internal noise of the receiving coil, the design and testing of such a sensor is described in detail. Results indicate that the primary field of the transmitting coil depends on the diameter, mass, and power of the coil. A higher mass–power product and a larger diameter causes a stronger primary field. Reducing the number of turns and increasing the clamp voltage reduces the switch-off time of the transmitting current effectively. Increasing the cross-section of the wire reduces the power consumption, but greatly increases the coil’s weight. The study of the receiving coil shows that the internal noise of the sensor is dominated by the thermal noise of the damping resistor. Reducing the bandwidth of the system and increasing the size of the coil reduces the internal noise effectively. The cross-sectional area and the distance between the sections of the coil have little effect on the internal noise. A less damped state can effectively reduce signal distortion. Finally, a portable TEM sensor with both a transmitting coil (constructed with a diameter, number of turns, and transmitting current of 0.5 m, 30, and 5 A, respectively) and a receiving coil (constructed with a length and resonant frequency of 5.6 cm and 50 kHz, respectively) was built. The agreement between experimental and calculated results confirms the theory used in the sensor design. The responses of an 82 mm mortar shell at different distances were measured and inverted by the differential evolution (DE) algorithm to verify system performance. Results show that the sensor designed in this study can not only detect the 82 mm mortar shell within 1.2 m effectively but also locate the target precisely.

## 1. Introduction

Unexploded ordnance (UXO) is an increasingly serious international humanitarian and environmental problem [1]. UXO causes many civilian casualties every year. Various geophysical methods have been used for UXO detection [2]. As a time-domain electromagnetic induction (EMI) detection method, TEM operates in the frequency range from tens to hundreds of kilohertz. In this range, the ground is almost transparent. It has emerged as an alternative technique to magnetometry [3] and ground-penetrating radar [4,5]. 

Generally, the UXO cleanup process consists of three stages: detection, inversion, and classification [5]. Detection is the inspection of the ground to determine the presence or absence of a UXO-like object. A great deal of progress has been made in this area to increase detection probability. One technique involves the use of a specially designed series of sophisticated ultrawideband sensors.

Since 1998, Oak Ridge National Laboratory (ORNL) has developed a helicopter-borne TEM system (ORAGS-TEM) for the detection of UXO at very low altitudes [6,7]. The transmitting coil of the ORAGS-TEM system is wrapped four times around a 12 m × 3 m rectangular loop, and the multiple-turn receiving coil is designed to have an area of 23 cm × 60 cm. The system can detect targets as small as 81 mm mortar rounds and 60 mm illumination rounds when flying 1–2 m above the ground. However, for sharp field decay [8], only very large targets can be reliably detected at survey heights of 3–4 m.

Meanwhile, a wide range of vehicle systems, with multi-axis transmitters and receivers, have been developed under SERDP-ESTCP programs [9]. These include MetalMapper [10], BUD [11], and TEMTADS [12]. The peak moments for MetalMapper and BUD reach up to 120 Am^2^ and 600 Am^2^, respectively. The TEMTADS sensor consists of 25 independent units arranged in a 5 × 5 array. Each unit consists of a transmitting coil of dimensions 35 cm × 35 cm and a receiving coil of dimensions 25 cm × 25 cm. These advanced EMI sensors provide measurements with a very high spatial diversity and wide dynamic range. However, they are large and heavy and cannot be readily used in rough or tree-covered terrains that do not allow vehicular access [13].

Compared with vehicular systems, portable systems can work in a variety of complex conditions. Developed by G&G Sciences (Grand Junction, CO, USA), the Man-Portable Vector (MPV) is an ultrawideband time-domain portable EMI instrument that measures all three components of the secondary field at five different locations [13,14]. However, limited by size, quality, and power consumption, the diameter of the transmitting coil is only 0.5 m and its peak moment is 27 Am^2^. This limits the performance of the system. Thus, it is critical to design the sensor under these constraints for portable systems. The design and optimization of the sensor has been widely discussed in airborne TEM systems. Ben K. Sternberg et al. have discussed the equivalent circuit model of the transmitting coil [15]. In addition to electrical parameters, parameters such as the size, weight, power consumption, turn-off time and cross-area of the transmitting coil will also be discussed here. Dehmel considers amplifier and thermal noise in his analysis of high-permeability cored coils, but only considers the thermal noise of the DC resistor in his analysis of an air-core coil within weight constraints [16]. For a TEM sensor under critical damping, the thermal noise of the damping resistor and the noise of the preamplifier will also be discussed in detail. Chen-Chen et al. [17,18] argue that the common-mode noise induced in the receiving coil can be significantly reduced by a differential structure. Chen-Shudong et al. [19] indicate that a receiving coil with greater radius and lower resonant frequency will result in a lower internal noise.

A high-performance portable TEM sensor for UXO detection is proposed here. The rest of the paper is organized as follows: With the physical structure and equivalent circuit proposed, the primary field along the axis of the transmitting coil is calculated and the influence of technical specifications such as diameter, mass, power, switch-off time, and current density is analyzed. Then, the internal noise of the receiving coil with a preamplifier and damping resistor is calculated and analyzed to determine the diameter and resonant frequency of the receiving coil. Finally, an experimental model is developed and parameters for both transmitting and receiving coils are measured to confirm the theory used in the sensor design. In addition, the responses of an 82 mm shell are measured to verify the performance of the system.

## 2. Structure and Equivalent Electrical Model of the Sensor

In this section, the physical structure with both transmitting and receiving coils is illustrated, followed by the equivalent electrical model. The electrical parameters are then calculated for further study.

### 2.1. Structure and Electrical Model of the Sensor

**Figure 1:**
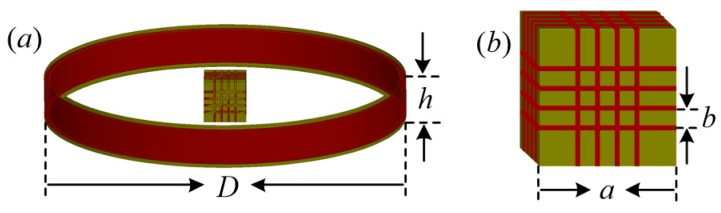
(**a**) Portable sensor with both transmitting and receiving coils; (**b**) three-component receiving coil.

The portable TEM sensor with both transmitting and receiving coils is shown in Figure 1.

As shown in Figure 1, the single-layer transmitting coil is wrapped around a round framework. The three-component receiving coil is wound on a square framework.

**Figure 2:**
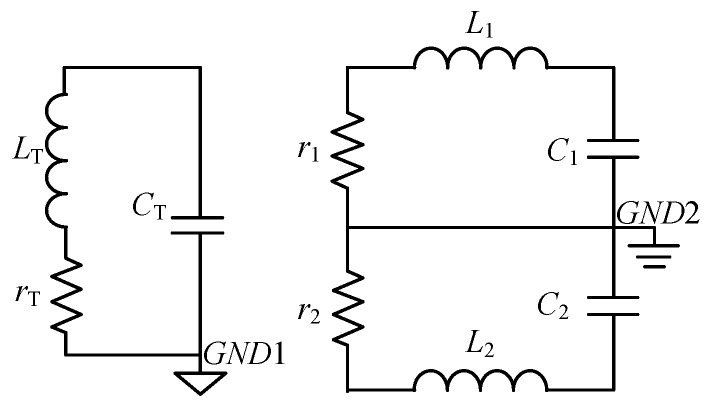
Schematic of the portable sensor.

Parameters *D*, *h*, *a*, and *b* denote the diameter, height of the transmitting coil, length of the receiving coil, and distance between the coil sections, respectively. The equivalent electrical model of the sensor described in Figure 1 is shown in Figure 2. Only the x-component receiving coil is illustrated.

In Figure 2, *L*_T_, *r*_T_, and *C*_T_ are the inductance, resistance, and capacitance of the transmitting coil, respectively. *L*_1_ = *L*_2_, *r*_1_ = *r*_2_, and *C*_1_ = *C*_2_ are the inductance, resistance, and capacitance of the receiving coil, respectively. Of all the parameters in Figure 2, resistance and inductance can be well predicted. However, stray capacitance, which results from several electrical couplings, is difficult to estimate. The following section provides an estimate of the electrical parameters of the sensor.

### 2.2. Electrical Parameter Estimation of the Transmitting Coil

The parameters *L*_T_ and *r*_T_ of the transmitting coil will be estimated here. Additionally, parameters such as mass *M*_T_, power consumption *P*_T_, and primary field *H*^P^ are also calculated.

(a) Estimation of the coil’s DC resistance:

The coil’s DC resistance *r*_T_ is given by

(1) $$r_{T}=\frac{\pi Dn_{T}}{\sigma_{T}S_{T}}.$$

Here, *σ*_T_ is the conductivity of the wire, *n*_T_ is the number of turns, and *S*_T_ is the cross-sectional area of the wire.

(b) Determination of the coil’s self-inductance:

When the diameter *D* is much greater than the diameter of the wire *d*_T_, and the value of *h* is high, we estimate the inductance using the following expression [20]:

(2) $$L_{T}=\frac{\mu_{0}Dn^{2}}{2}\left[\ln\left(\frac{4D}{h}\right)-0.5\right].$$

Here, *μ*_0_ is the permeability of vacuum.

(c) Determination of the coil’s mass:

The mass of the transmitting coil is an important parameter, especially for portable systems. It can be estimated by the following expression:

(3) $$M_{T}=\rho_{T}n_{T}D\pi S_{T}.$$

Here, *ρ_T_* is the density of the wire.

(d) Determination of the power consumption of the coil:

The coil’s power consumption *P*_T_ is determined by the transmitting current and resistance. It can be calculated as follows:

(4) $$P_{T}=\frac{\pi Dn_{T}I_{T}^{2}}{\sigma_{T}S_{T}}.$$

Here, *I*_T_ is the transmitting current of the sensor.

(e) Primary field of the sensor:

The primary field is determined by the diameter, number of turns, transmitting current, and relative position *R* of the target. To simplify the analysis, we will only calculate the primary field along the coil axis, which is given by

(5) $$H^{P}=\frac{n_{T}I_{T}D^{2}}{{\left(4R^{2}+D^{2}\right)}^{3/2}}.$$

Based on the electrical model and the parameters calculated above, the design and optimization of the transmitting coil will be described in the next section.

### 2.3. Electrical Parameter Estimation of the Receiving Coil

All parameters of the receiving coil in Figure 2 will be estimated. Additionally, parameters such as resultant area *S*_r_ and resonant frequency *f*_0_ are calculated.

(a) Estimation of the coil’s DC resistance:

The coil’s DC resistance *r*_1_ is given by

(6) $$r_{1}=\frac{8an_{r}}{\pi \sigma_{r}d_{r}^{2}}.$$

Here, *σ_r_* is the conductivity of the wire, *n_r_* is the number of turns, and *d_r_* is the diameter of the wire used for the receiving coil.

(b) Determination of the coil’s self-inductance:

For an air-core induction coil, when the coil length *a* is much greater than the cross-sectional length *d,* we estimate the self-inductance *L*_0_ for one section, and mutual inductance *M* between sections, using the following expression [20]:

(7) $$L_{0}=\frac{\mu_{0}an_{r}^{2}}{8\pi }\left[\ln\left(\frac{a}{d}\right)-0.3782\right],$$

(8) $$M=\frac{\mu_{0}an_{r}^{2}}{8\pi }\left[\ln\left(\frac{1+\sqrt{1+\gamma }}{1+\sqrt{2+\gamma }}\sqrt{\frac{2}{\gamma }+1}\right)+\left(\sqrt{2+\gamma }-2\sqrt{1+\gamma }+\sqrt{\gamma }\right)\right].$$

Here, *γ* = *b*^2^/*a*^2^, where *b* is the distance between two sections. The total induction of the coil includes both self-inductance and mutual inductance.

(9) $$L_{1}=2L_{0}+M_{12}+M_{13}+M_{14}+M_{23}+M_{24}+M_{34}$$

Here, *M*_12_ = *M*_23_ = *M*_34_, *M*_13_ = *M*_24_ and *M*_14_ are the mutual inductance of the coil.

(c) Determination of the coil’s capacitance:

According to Seran [21], the total capacitance can be divided into two types, self-capacitance *C_A_* and section-to-section capacitance *C_B_*.

(10) $$C_{A}=\frac{C_{a}}{N}$$

(11) $$C_{B}=\frac{4\left(N-1\right)C_{b}}{N^{2}}$$

Here, *C_a_* is the self-capacitance of one section and *C_b_* is the capacitance between two sections. The capacitance *C*_1_ in Figure 2 can be calculated as follows:

(12) $$C_{1}=2\left(C_{A}+C_{B}\right).$$

(d) Determination of the coil’s resultant area:

The coil’s resultant area can be calculated as follows:

(13) $$S_{r}=a^{2}n_{r}.$$

(e) Resonant frequency of the coil *f_r_*:

The resonant frequency is determined by the inductance and capacitance of the coil, which is given by

(14) $$f_{r}=\frac{1}{2\pi \sqrt{L_{1}C_{1}}}.$$

Based on the electrical model and the parameters calculated above, the determination of the sensor parameters will be described in the following sections.

## 3. Design of the Transmitting Coil

According to the design requirements, the strength of the primary field should be 50% greater than that of the MPVII [14]. The switch-off time of the transmitting current should be no more than 20 μs. The current density of the wire should not exceed 5 A/mm^2^. The relationship between the primary field and the parameters of the transmitting coil will be discussed in detail.

### 3.1. Determination of Diameter and Mass–Power Product of the Transmitting Coil

According to Equations (3)–(5), the primary field along the axis of the transmitting coil is calculated with the mass, power consumption, and diameter of the coil.

(15) $$H^{P}=\frac{1}{\pi }\sqrt{\frac{\sigma_{T}}{\rho_{T}}}\frac{\sqrt{M_{T}P_{T}}}{D^{2}{\left(4{R^{2}/D}^{2}+1\right)}^{3/2}}$$

**Figure 3:**
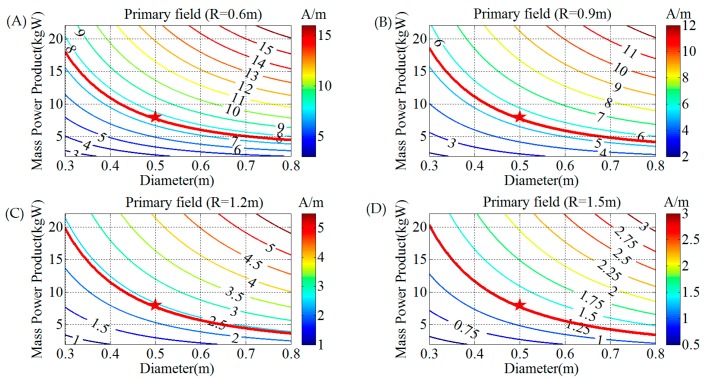
Contour map of the primary field *H*^P^ versus diameter *D* and mass–power product *M_T_P_T_* for different distances *R*. (**A**) Primary field *H*^P^ with distance *R* = 0.6 m, (**B**) primary field *H*^P^ with distance *R* = 0.9 m, (**C**) primary field *H*^P^ with distance *R* = 1.2 m, (**D**) primary field *H*^P^ with distance *R* = 1.5 m.

Using Equation (15), the contour maps of the primary field for the transmitting coil are shown in Figure 3, in which the value of the primary field *H*^P^ depends on the diameter *D* and mass–power product *M_T_P_T_* when distance *R* = 0.6, 0.9, 1.2, and 1.5 m, respectively.

As shown in Figure 3, the primary field *H*^P^ decreases rapidly with the diameter, and increases rapidly with the mass–power product for any distance. The red line in Figure 3 represents the primary field 50% greater than that of the MPVII at different distances. The mass–power product *M*_T_*P*_T_ and diameter *D* corresponding to the area above the red line satisfy the conditions; this means that a greater mass–power product with a larger diameter can achieve a higher primary field. 

Taking into account the restrictions of the coil diameter, power, and mass for a portable system, the values 0.5 m and 8 kgW are chosen for the diameter and mass–power product, respectively, as shown by the red stars in Figure 3. We will further determine parameters such as the number of turns, transmitting current, and cross-sectional area of the wire.

### 3.2. Determination of Turns and Current of the Transmitting Coil

For constant voltage clamp technology, the switch-off time *t_off_* can be calculated as

(16) $$t_{off}=\frac{L_{T}I_{T}}{V_{dss}}.$$

Here, *V_dss_* is the clamping voltage. According to Equations (2)–(4) and (16):

(17) $$t_{off}=\frac{\mu_{0}}{2\pi }\left[\ln\left(\frac{4D}{h}\right)-0.5\right]\sqrt{\frac{\sigma_{T}}{\rho_{T}}}\sqrt{M_{T}P_{T}}\frac{n_{T}}{V_{dss}}.$$

**Figure 4:**
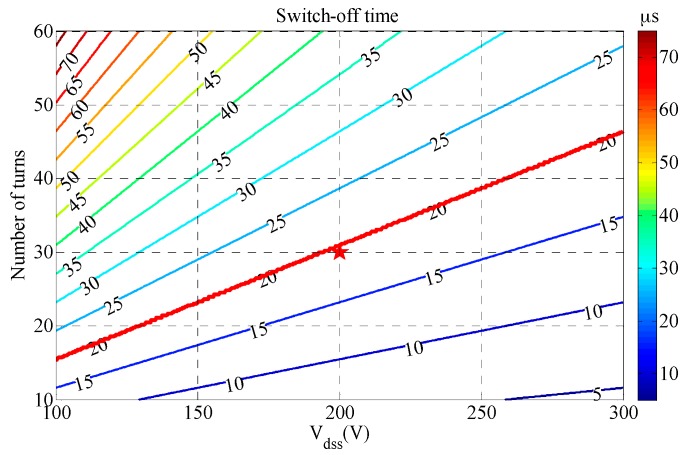
Contour map of the switch-off time *t_off_* versus *V_dss_* and number of turns.

The contour map of the switch-off time for the transmitting coil obtained using Equation (17) is shown in Figure 4, in which the value of *t_off_* depends on those of the clamping voltage *V_dss_* and the number of turns when *D* = 0.5 m, *h* = 7 cm, and *M_T_P_T_* = 8 KgW.

As shown in Figure 4, the switch-off time decreases rapidly with *V_dss_* and increases rapidly with the number of turns. The red line in Figure 4 indicates that the switch-off time equals 20 µs. Coil turns *n*_T_ and *V_dss_* corresponding to the area below the red line satisfy the conditions, which means that a larger *V_dss_* with a smaller number of turns can reduce the switch-off time. Finally, the *V_dss_* is chosen to be 200 V and the number of turns is determined to be 30.

According to Equations (5) and (6),

(18) $$n_{T}I_{T}=\frac{1}{\pi }\sqrt{\frac{\sigma_{T}}{\rho_{T}}}\frac{1}{D}\sqrt{M_{T}P_{T}}.$$

When the number of turns is 30, the transmitting current is calculated as 5 A.

### 3.3. Determination of the Cross-Sectional Area of Wire for the Transmitting Coil

Due to safety considerations, the current density *I*_d_ of the wire should be no greater than 5 A/mm^2^.

(19) $$I_{d}=\frac{I_{T}}{S_{T}}$$

**Figure 5:**
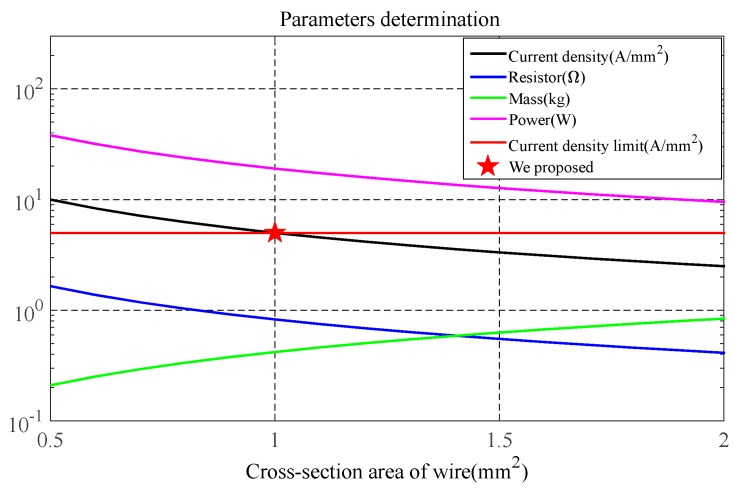
Parameters of current density, resistance, mass, and power consumption of the transmitting coil versus the cross-sectional area of the wire.

The parameters such as current density, resistance, mass, and power consumption of the transmitting coil obtained by Equations (1), (3), (4) and (19) are shown in Figure 5 with respect to the cross-sectional area of the wire.

As shown in Figure 5, both the current density and resistance decrease with the cross-sectional area of the wire while the mass increases. The red line in Figure 5 represents the current density limitation. A current density *I*_d_ corresponding to the area below the red line satisfies the conditions.

Finally, the cross-sectional area of the copper wire is chosen to be 1 mm^2^ and the current density is calculated as 5 A/mm^2^. Correspondingly, the DC resistance, height, inductance, mass, and power consumption of the transmitting coil are 0.83 ohms, 7 cm, 806 μH, 0.42 kg, and 19.1 W, respectively. With the transmitting coil determined, the design of the receiving coil will be discussed in detail in the next section.

## 4. Design of the Receiving Coil

**Figure 6:**
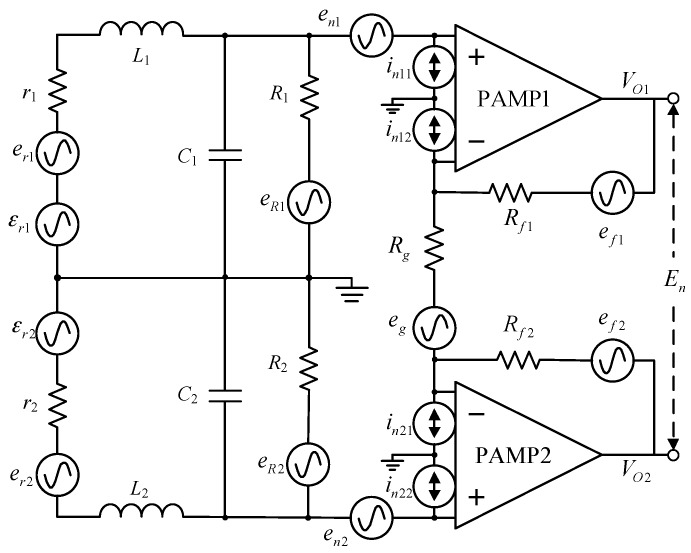
Sensor circuit with signal and equivalent noise locations.

A central-tapped air-core coil combined with a differential preamplifier is chosen to suppress the common-mode noise induced in exploration surveys. The signal and internal noise of the receiving coil and preamplifier are shown in Figure 6.

In Figure 6, *r*_1_ = *r*_2_, *L*_1_ = *L*_2_, *C*_1_ = *C*_2_, and *R*_1_ = *R*_2_ are the resistance, inductance, capacitance, and damping resistance, respectively, of the receiving coil in the differential mode. *R_g_* and *R_f_*_1_ = *R_f_*_2_ are the gain resistance and feedback resistance of the preamplifier, respectively. With respect to the sensor, the induced voltage is denoted by *ɛ*_r1_, *ɛ*_r2_, the output voltage by *V*_O1_, *V*_O2_, and the total output noise by *E*_n_. In this section, the bandwidth and noise characteristics of the receiving coil will be discussed in detail.

### 4.1. Bandwidth of the Sensor

According to Figure 6, the transmission characteristics *H (ω)* of the receiving coil are calculated as follows:

(20) $$H\left(\omega \right)=\frac{V_{O1}}{\varepsilon_{r1}}=\frac{G\omega_{0}^{2}}{{\left(j\omega \right)}^{2}+2\zeta \omega_{p}\left(j\omega \right)+\omega_{p}^{2}}.$$

Here, *G* = (2*R_f_*_1_**/***R_g_*) + 1 is the gain of the preamplifier, *ω*_0_ = 2π*f*_0_, and *ω*_p_ = *ω*_0_(1 + *r*_1_/*R*_1_)^1/2^. The symbol ζ denotes the damping coefficient, defined as

(21) $$\zeta =\frac{R_{1}r_{1}C_{1}+L_{1}}{2\sqrt{L_{1}C_{1}R_{1}\left(r_{1}+R_{1}\right)}}.$$

The amplitude–frequency characteristics of the receiving coil can be calculated as follows:

(22) $$|H(f)|=\frac{G}{\sqrt{{\left(f/f_{0}\right)}^{4}+2{\left(f/f_{0}\right)}^{2}\left(1+r_{1}/R_{1}\right)\left(2\zeta^{2}-1\right)+{\left(1+r_{1}/R_{1}\right)}^{2}}}.$$

The cut-off frequency *f*_b_ of the coil is defined as

(23) $$\frac{\left|H(0)\right|}{\left|H(f_{b})\right|}=\sqrt{2}.$$

By Equations (22) and (23), the bandwidth *BW*_C_ of the receiving coil is

(24) $$BW_{c}=f_{b}=f_{0}\sqrt{1+\frac{r_{1}}{R_{1}}}\sqrt{1-2\zeta^{2}+\sqrt{4\zeta^{4}-4\zeta^{2}+2}}.$$

As the damping resistance is much greater than the coil’s DC resistance, Equation (24) can be simplified as

(25) $$BW_{c}=f_{0}\sqrt{1-2\zeta^{2}+\sqrt{4\zeta^{4}-4\zeta^{2}+2}}.$$

**Figure 7:**
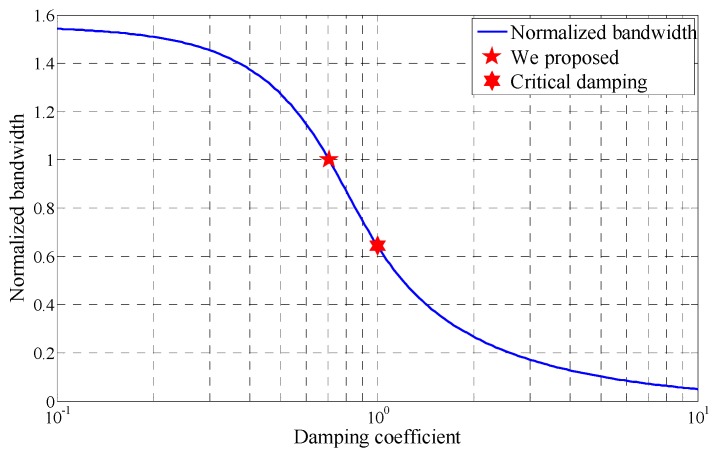
Bandwidth of the receiving coil with different damping coefficients.

The normalized bandwidth of the receiving coil obtained by Equation (25) is shown in Figure 7, in which the value of *BW*_C_/*f*_0_ depends on the damping coefficient.

Figure 7 shows that the bandwidth of the receiving coil decreases with the damping coefficient. The −3 dB bandwidth is about 64% of the resonant frequency in the critical damping state. To reduce signal distortion, a less damped state is chosen where the damping coefficient is set as $\sqrt{2}/2$, and the bandwidth is equal to the resonant frequency.

### 4.2. Internal Noise of the Sensor

According to Figure 6, the total output noise *E_n_* consists of three components: the thermal noise of the resistors, and the voltage, and the current noise of the preamplifier. Assuming uncorrelated noise sources, the total noise PSD $E_{n}^{2}$ is

(26) $$E_{n}^{2}=2E_{nr_{1}}^{2}+2E_{nR_{1}}^{2}+E_{nR_{g}}^{2}+2E_{nR_{f_{{}_{1}}}}^{2}+2E_{ne_{1}}^{2}+2E_{ni_{11}}^{2}+2E_{ni_{12}}^{2}.$$

Here, $E_{nr_{1}}^{2}$, $E_{nR_{1}}^{2}$, $E_{nR_{g}}^{2}$, $E_{nR_{f_{1}}}^{2}$, $E_{ne_{1}}^{2}$, and $E_{ni_{11}}^{2}$ are the output noise PSDs of the DC resistance, damping resistance, gain resistance, feedback resistance, voltage, and current noise of the preamplifier, respectively.

According to Equation (26), the equivalent noise PSD at the preamplifier input can be obtained by normalizing the output PSD $E_{n}^{2}$ of the amplifier when ζ$=\sqrt{2}/2$:

(27) $$\frac{E_{n}^{2}}{G^{2}}=\frac{2\omega_{0}^{4}}{\left(\omega^{4}+\omega_{p}^{4}\right)}\left[e_{r_{1}}^{2}+\left(\frac{e_{R_{1}}^{2}}{R_{1}^{2}}+i_{n_{{}_{11}}}^{2}\right)\left(\omega^{2}L_{1}^{2}+r_{1}^{2}\right)\right]+2e_{n_{1}}^{2}+e_{R_{g}}^{2}+\frac{2}{G^{2}}\left(e_{R_{f_{1}}}^{2}+i_{n_{11}}^{2}R_{f_{1}}^{2}\right).$$

According to Chen-Shudong et al. [17], the thermal noise of the damping resistor dominates the total noise PSD at the input of the preamplifier. In this case, Equation (27) can be simplified as

(28) $$\frac{E_{n}^{2}}{G^{2}}=\frac{2e_{R_{1}}^{2}}{R_{1}^{2}}\frac{\omega_{0}^{4}\left(\omega^{2}L_{1}^{2}+r_{1}^{2}\right)}{\left(\omega^{4}+\omega_{p}^{4}\right)}.$$

The equivalent input noise power of the amplifier $V_{n}^{2}$ can be obtained by integrating Equation (28) in the range [0, *f*_s_], where *f*_s_ is the bandwidth of the system. With *f_s_* set as *f*_0_, the equivalent input noise power of the amplifier $V_{n}^{2}$ can be calculated as

(29) $$V_{n}^{2}=\int_{0}^{f_{s}}\frac{E_{n}^{2}}{G^{2}}df=\frac{4\sqrt{2}kT}{\pi C_{1}}\int_{0}^{1}\frac{{\left(f/f_{s}\right)}^{2}}{\left({\left(f/f_{s}\right)}^{4}+1\right)}df/f_{s}\approx \frac{\sqrt{2}kT}{\pi C_{1}}.$$

According to Equations (7)–(9), (13) and (29), the normalized noise to the resultant area *S_r_* of the receiving coil is given as follows:

(30) $$V_{nS}=\frac{\sqrt{V_{n}^{2}}}{S_{r}}=\sqrt{2kT\pi \lambda }\frac{f_{0}}{a^{1.5}}.$$

Here, $\lambda =16L_{1}/(an^{2})$, which only depends on the ratios *a*/*d* and *b*^2^/*a*^2^.

As shown in Equation (30), the internal noise *V_nS_* of the receiving coil depends on four factors: coil resonance frequency *f*_0_, coil length *a*, distance between the sections *b*, and section length *d*.

### 4.3. Determination of the Sensor Parameters

#### 4.3.1. Determination of Length and Resonant Frequency

**Figure 8:**
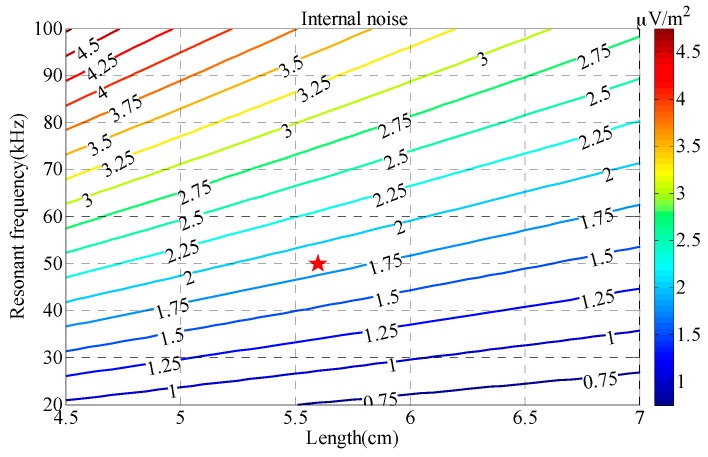
Contour map of the normalized internal noise *V_nS_* versus length *a* and resonant frequency *f*_0_.

The contour map of the sensor’s internal noise *V_nS_* obtained by Equation (30) is shown in Figure 8, in which the value of *V_nS_* depends on those of *a* and *f*_0_ when *d* = 4 mm and *b* = 10 mm.

As shown in Figure 8, the sensor’s internal noise *V_nS_* decreases rapidly with length *a* and increases rapidly with resonant frequency *f*_0_; this means that a larger length with a lower resonant frequency can achieve a lower level of sensor internal noise. Finally, the length of the receiving coil is set to 5.6 cm and the resonant frequency is set to 50 kHz to reduce the internal noise of the receiving coil.

#### 4.3.2. Determination of the Cross-Section Radius

**Figure 9:**
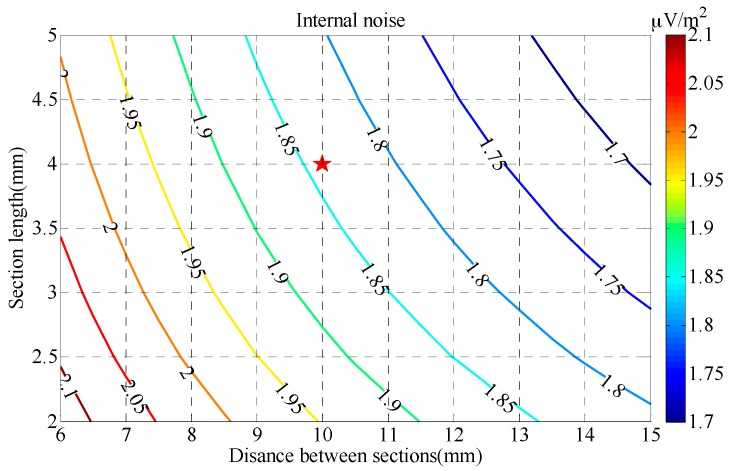
Contour map of the normalized sensor internal noise *V_nS_* versus distance between sections *b* and section length *d*.

The contour map of the sensor internal noise *V_nS_* obtained by Equation (30) is shown in Figure 9, in which the value of *V_nS_* depends on the values of the distance between sections *b* and the section length *d* when *f*_0_ = 50 kHz and *a* = 5.6 cm.

In Figure 9, the sensor’s internal noise *V_nS_* decreases slowly with both the distance between sections and the section length, which means that a larger distance between sections and a larger section length can lower the sensor internal noise. The distance between sections *b* and the section length *d* are therefore designed as 10 mm and 4 mm, respectively.

Finally, the length *a*, resonant frequency *f*_0_, distance between sections *b*, and section length *d* are chosen to be 5.6 cm, 50 kHz, 10 mm, and 4 mm, respectively. The detailed parameters of the sensor are given in Section 5.

## 5. Parameter Testing

**Figure 10:**
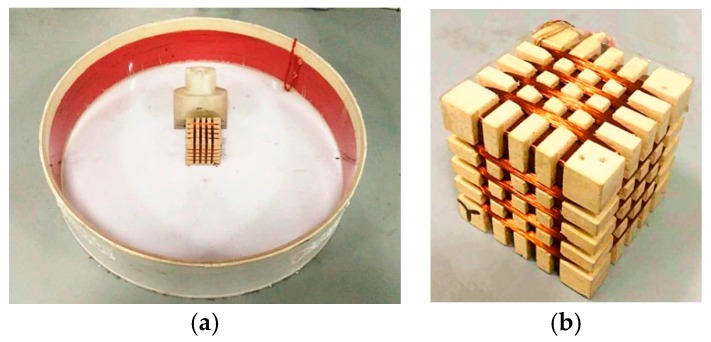
(**a**) Transmitting coil of portable TEM sensor, and (**b**) three-component receiving coil.

According to the discussion in Section 3 and Section 4, an experimental model of a portable TEM sensor with both a transmitting coil and a receiving coil was constructed, as shown in Figure 10.

A low-noise operational amplifier LT6234 (Linear Technology Corporation, Milpitas, CA, USA) was chosen to amplify the induced voltage. The specifications of the sensor are listed in Table 1 and Table 2.

Based on the parameters in Table 1 and Table 2, the transmitting current, transmission characteristics, and normalized power spectrum of the receiving coil were measured.

### 5.1. Transmitting Current Testing

**Figure 11:**
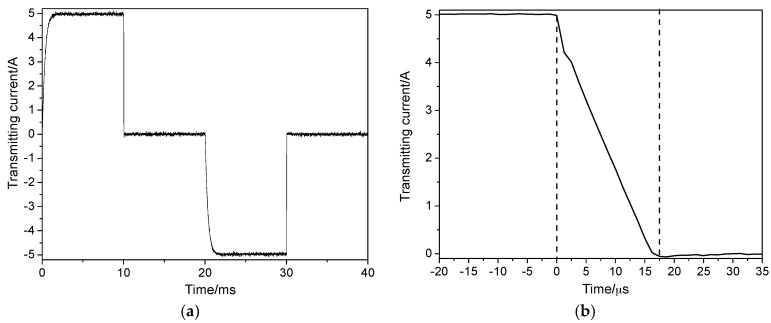
(**a**) Transmitting current waveform of the portable sensor; (**b**) switch-off time of the transmitting current.

Transmitting current was measured using a GWINSTEK GDS-3502 digital oscilloscope (Good Will Instrument Co., Ltd., Taiwan, China), as shown in Figure 11.

As can be seen from Figure 11a, the period, duty cycle, and amplitude of the transmitting current are 40 ms, 50%, and 5.0 A, respectively. In Figure 11b, the transmitting current decreases linearly and the switch-off time approaches 20 µs. Both the amplitude and switch-off time of the transmitting current are consistent with the theoretical values.

### 5.2. Transmission Characteristics Testing

**Figure 12:**
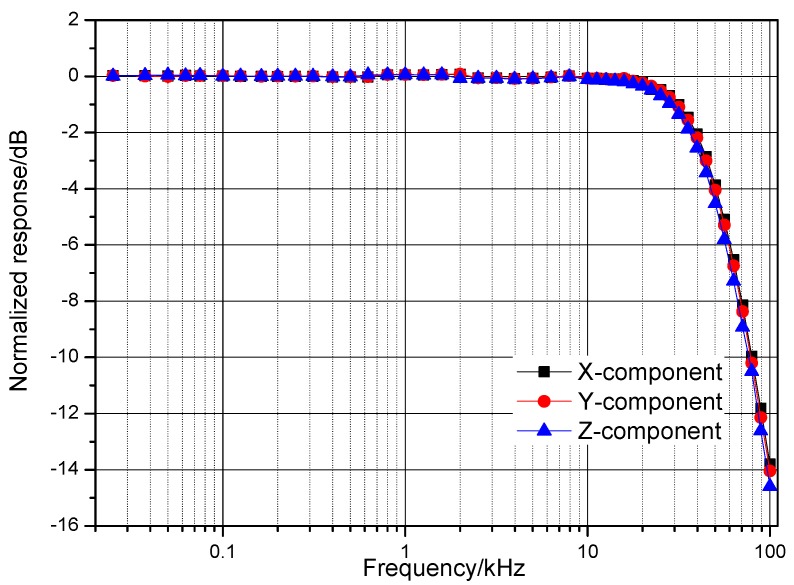
Normalized transmission characteristics of the receiving coil.

The transmission characteristics of the three-component receiving coils were measured using an Advantest R9211E digital spectrum analyzer (Advantest, Tokyo, Japan). The normalized transmission characteristics are shown in Figure 12.

As shown in the preceding figure, the normalized response amplitude is flat as long as the frequency is less than the self-resonant frequency, which is about 50 kHz. Above the self-resonant frequency, the response amplitude of the three-component receiving coil decreases rapidly. The transmission characteristics of the three-component receiving coil are highly uniform.

### 5.3. Internal Noise Testing

**Figure 13:**
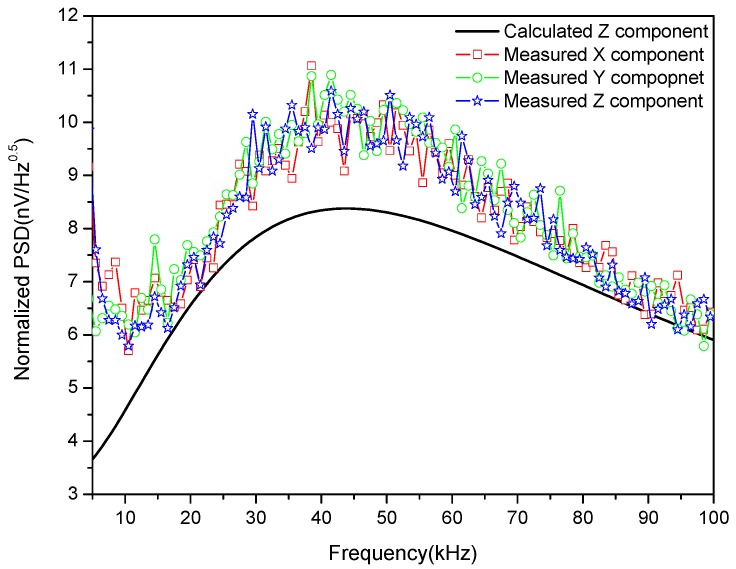
Comparison of calculated and measured PSD of the sensor.

Based on the parameters in Table 2, the equivalent noise PSD at the preamplifier’s input in Equation (27) was calculated and compared with that measured using an Advantest R9211E digital spectrum analyzer (Advantest, Tokyo, Japan), as shown in Figure 13.

As shown in Figure 13, both the measured and calculated PSD of the normalized noise first increase with frequency and then decrease, reaching a maximum at the resonant frequency. The maximum value of the measured results is about $10nV/\sqrt{Hz}$, while the calculated result gives $8nV/\sqrt{Hz}$. The difference between the two mainly comes from the electromagnetic interference that may not be completely eliminated by the shielding room, especially at low frequencies.

## 6. Field Experiment

**Figure 14:**
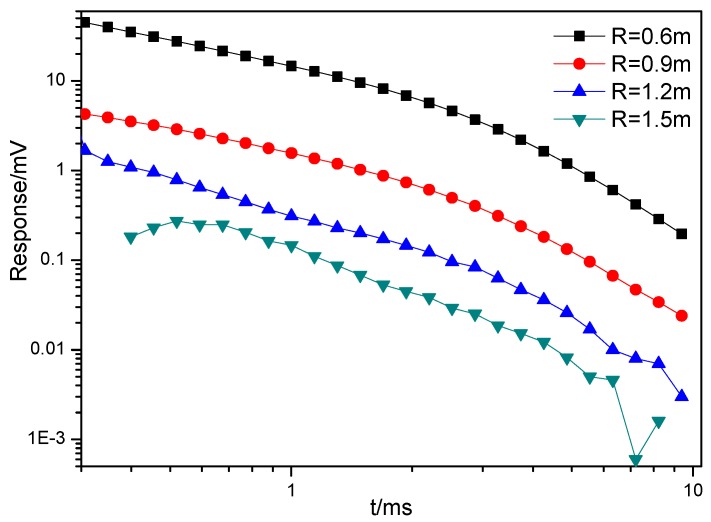
Responses of an 82 mm mortar shell at different distances.

Field experiments were conducted to test the performance of the portable sensor. They were carried out at the Central Campus of Jilin University. An 82 mm mortar shell was chosen as the target and the distance in Equation (5) was set to 0.6 m, 0.9 m, 1.2 m, and 1.5 m. The responses of the 82 mm mortar shell were measured in 60 s at each distance. The responses are shown below:

As can be seen from Figure 14, the amplitude of the response decreases rapidly with distance between the target and the sensor. The response can be accurately recorded when the distance is not greater than 1.2 m. When the distance reaches 1.5 m, the signal-to-noise ratio drops rapidly, and the early response and later response are completely distorted. Therefore, the detection distance for an 82 mm mortar shell is no less than 1.2 m.

**Figure 15:**
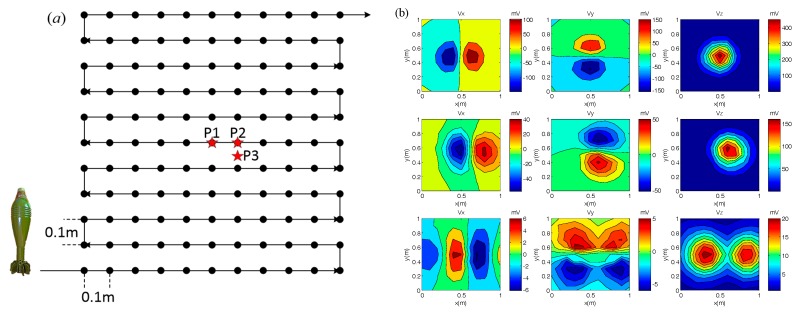
(**a**) Measurement for field experiment; (**b**) responses of 82 mm mortar shell.

Additional experiments were done to further verify the performance of the system. The measurement diagram and the three-component responses of the 82 mm mortar shell are as follows:

As shown in Figure 15a, the measurement district is 1 m × 1 m, and the distance between the points and lines is 0.1 m. Figure 15b shows the three-component responses of an 82 mm mortar shell at different positions (P1, P2, and P3) with different orientations. The difference in target response is clearly recorded by the sensor.

Parameters such as position (x, y, z) and orientation (θ, Φ) of the target calculated by DE algorithm [22] were compared to the measured ones. The results are as follows:

As shown in Table 3, the calculated parameters are in good agreement with the measured ones. The maximum error comes from θ when θ = 0°. In this paper, the parameter θ is estimated according to cos θ. When θ = 8°, cos θ is 0.99, which is very close to 1, where θ = 0°. Thus, the calculated parameter θ will be seriously disturbed by noise around 0°. The inversion algorithm needs to be further optimized, but overall, the response measured by the sensor can reflect the target’s position and orientation accurately.

## 7. Conclusions and Prospects

A high-performance portable transient electromagnetic sensor with both transmitting and receiving coils for UXO detection was designed, built, and tested to improve performance in exploration surveys. The design and testing of the sensor were described in detail.

The primary field along the axis of the transmitting coil was calculated based on the diameter and mass–power product of the coil. The results showed that the primary field increases with both the diameter and mass–power product. The switch-off time of the transmitting current can be reduced by increasing the clamping voltage or decreasing the number of turns. Increasing the cross-sectional area of the wire reduces the power consumption, but increases the coil’s mass.

The bandwidth and internal noise model of the receiving coil with a damping resistor and a preamplifier was established and analyzed. Results showed that the thermal noise of the damping resistor dominates the sensor’s internal noise. To realize a high-sensitivity sensor, the length of the receiving coil should be as large as possible and the resonant frequency should be as small as possible. The section size has little effect on the sensor’s internal noise. Besides this, a less-damped state will effectively reduce signal distortion.

Finally, the transmitting coil was constructed with a diameter, number of turns, current, and cross-sectional area of 0.5 m, 30, 5 A, and 1 mm^2^, respectively. The receiving coil was constructed with a length, resonant frequency, distance between sections, and section length of 5.6 cm, 50 kHz, 10 mm, and 4 mm, respectively. Experiments were conducted to test the sensor’s performance for both transmitting and receiving coils. The conformity between the experimental and calculated results confirmed the theory used in the sensor design. Field experiments show that the sensor designed in this manuscript can not only detect the 82 mm mortar shell at a distance of up to 1.2 m but also determine the target’s information, such as position and orientation, accurately.

## Figures and Tables

**Table 1:** Parameters of transmitting coil.

Parameters	Symbol	Value
Diameter (cm)	*D*	50
High (cm)	*h*	7
Number of turns	*n_T_*	30
Section area of wire (mm^2^)	*S_T_*	1
DC resistance (Ω)	*r_T_*	0.9
Inductance (µH)	*L_T_*	790

**Table 2:** Parameters of three-component receiving coil.

Parameters	Symbol	Value
Length (mm)	*a*	56
Section length (mm)	*d*	4
Distance between sections (mm)	*b*	10
Inductance (mH)	*L*_1__x_ *= L*_2__x_, *L*_1__y_ *= L*_2__y_, *L*_1__z_ *= L*_2__z_	10.5, 10.7, 11.0
DC resistance (Ω)	*r*_1__x_ *= r*_2__x_, *r*_1__y_ *= r*_2__y_, *r*_1__z_ *= r*_2__z_	50.9, 50.7, 51.0
Capacitance (pF)	*C*_1__x_ *= C*_2__x_, *C*_1__y_ *= C*_2__y_, *C*_1__z_ *= C*_2__z_	1004.6, 1005.2, 1007.4
Resonant frequency (kHz)	*f*_0__x_, *f*_0__y_, *f*_0__z_	49.0, 48.5, 47.8
Damping Resistance (Ω)	*R*_1__x_ *= R*_2__x_, *R*_1__y_ *= R*_2__y_, *R*_1__z_ *= R*_2__z_,	2320, 2320, 2320
Resistance of preamplifier (Ω)	*R_g_*, *R_f_*_1_ = *R_f_*_2_	100, 2000
Voltage noise of the amplifier ($nV/\sqrt{Hz}$)	*e_n_*_1_ = *e_n_*_2_	1.9
Current noise of the amplifier ($pA/\sqrt{Hz}$)	*i_n_*_11_ = *i_n_*_12_ = *i_n_*_21_ = *i_n_*_22_	0.78

**Table 3:** Inversions of measured data.

Measured					Calculated (m)				
x (m)	y (m)	z (m)	θ (°)	Φ (°)	x (m)	y (m)	z (m)	θ (°)	Φ (°)
0.5	0.5	−0.38	0	-	0.52	0.49	−0.35	8	-
0.6	0.5	−0.48	45	90	0.57	0.50	−0.50	46	89
0.6	0.45	−0.43	90	90	0.59	0.44	−0.42	91	93
